# Gene-edited healthy donor CAR T cells show superior anti-tumour activity compared to CAR T cells derived from patients with lymphoma in an in vivo model of high-grade lymphoma

**DOI:** 10.1038/s41375-021-01324-z

**Published:** 2021-06-18

**Authors:** Charlotte Elizabeth Graham, Agnieszka Jozwik, Ruby Quartey-Papafio, Nikolaos Ioannou, Ana M. Metelo, Carlo Scala, Glenda Dickson, Orla Stewart, Maria Almena-Carrasco, Elisa Peranzoni, Alan G. Ramsay, Piers E. M. Patten, Thomas Pertel, Farzin Farzaneh, Sandra Dupouy, Andrea Pepper, Reuben Benjamin

**Affiliations:** 1 Faculty of Life Sciences and Medicine, School of Cancer and Pharmaceutical Sciences, King’s College London, London, United Kingdom; 2 King’s College Hospital NHS Foundation Trust, London, United Kingdom; 3 Institut de Recherches Internationales Servier, Paris, France; 4 Allogene Therapeutics, San Francisco, CA United States; 5 Brighton and Sussex Medical School, University of Sussex, Brighton, United Kingdom

**Keywords:** Tumour immunology, Immunotherapy

## To the Editor:

CD19-targeted autologous chimeric antigen receptor (CAR) T-cell therapy has shown dramatic response rates in relapsed and refractory patients with B-cell malignancies [1–4]. However, a growing body of literature has demonstrated T-cell dysfunction in some cancer patients, which impairs the effectiveness of the end CAR T-cell product [5–7]. Healthy donor (HD) CAR T cells could potentially provide a source of more functional cells. Graft-versus-host disease (GvHD) limits the use of non-HLA-matched HD CAR T cells, but gene-editing to remove the native alpha beta T-cell receptor (TCR) from HD CAR T cells has allowed HD TCR^−^ CAR T cells to be given to HLA unmatched patients with B-acute lymphoblastic leukaemia or with B-cell lymphoma in clinical trials [8–10].

This study sought to compare the functionality of HD and HD TCR^−^ CAR T cells with B-cell lymphoma patient CAR T cells. In vitro functional assays were performed and in vivo activity was assessed using a xenograft model of high-grade B-cell lymphoma (NOD/SCID/IL2Rγ^null^(NSG) Raji luciferase), correlating efficacy with phenotypic features of the CAR T-cell product.

**Fig. 1:**
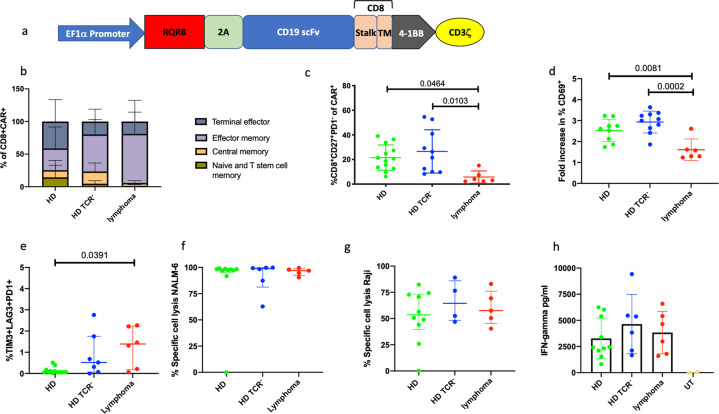
In vitro phenotypic and functional characteristics of CAR T cells from lymphoma patients and healthy donors. **a** Schematic diagram of CAR construct. **b** Memory subsets on CD8^+^CAR^+^ cells are shown. Naïve and stem cell memory (CD45RO^−^CCR7^+^), central memory (CD45RO^+^CCR7^+^), effector memory (CD45RO^+^CCR7^−^), terminal effector (CD45RO^−^CCR7^−^). Mean and standard deviation are displayed. **c** Percentage of CD8^+^CD27^+^PD-1^−^ cells are displayed for the different CAR T groups. They are seen at a higher proportion in HD and HD TCR^−^ CAR T cells than with lymphoma CAR T cells. Mean with standard deviation (SD) displayed, one-way ANOVA *p* = 0.0182, adjusted *p* values for paired comparisons using the Tukey’s multiple comparisons test. **d** Fold increase in CD69 is significantly higher on HD and HD TCR^−^ CAR T cells compared to lymphoma CAR T cells when stimulated with NALM-6 cells in a 1:1 ratio for 24 h (mean with SD) One-way ANOVA *p* = 0.0003, adjusted *p* values for paired comparisons displayed using the Tukey’s multiple comparisons test. **e** Percentage of CD8^+^ CAR T cells triple positive for PD-1, LAG3 and TIM3 is displayed (Kruskal Wallis *p* = 0.0277, with adjusted *p* values displayed using the Dunn’s multiple comparisons test). **f** CAR T or UT cells were cultured with GFP^+^ NALM-6 or **g** GFP^+^ Raji cells in a 1:1 ratio for 24 h and the percentage of specific cell lysis following 24 h co-culture is shown (median with interquartile range) (specific cell lysis = (% viable with UT-% viable with CAR T cells)/% viable with UT) × 100, all CAR T groups showed superior killing to UT cells. **h** IFNγ secretion as measured by Luminex platform in supernatant harvested from 24-h co-culture with NALM-6 cell line. IFNγ values are shown after removing the baseline values (cytokine secretion from CAR T cells cultured alone) from individual samples. All CAR T groups showed increased IFNγ production compared to UT cells (mean with SD).

Peripheral blood mononuclear cells were collected from consenting B-cell lymphoma patients (*n* = 7) who underwent apheresis for a commercial CAR T-cell product, and from young adult HDs (age 18–30 years, *n* = 13) (Human Tissue Authority Licence number 12223 and King’s College London Research Ethics Committee Ref: HR-17/18-5515). Young adults were chosen because aged T cells are thought to be more terminally differentiated with reduced proliferative capacity [11]. Using the same CAR construct (anti-CD19 4G7 scFv 4-1BB CD3ζ lentiviral CAR) (Fig. 1a) and manufacturing process, research grade lymphoma patient and HD CAR T cells were produced. HD TCR^−^ CAR T cells were made by electroporating HD CAR T cells with TALEN^®^ mRNA targeting the *TRAC* locus (TCR alpha constant). Residual unedited CAR T cells were removed by magnetic bead depletion [12]. Manufacturing lasted 14 days for all CAR T-cell groups prior to cryopreservation and storage.

Lymphoma patients were older (median age 52, range 30–73 years) than HDs, and met eligibility criteria for a commercial anti-CD19 CAR T-cell product. They had received a median of three lines of therapy (range 2–5, Table S1). 3/7 had a prior autologous haematopoietic stem cell transplant (HSCT) and 1/7 had a prior allogeneic HSCT. One of 7 patients had primary mediastinal B-cell lymphoma, 3/7 had diffuse large B-cell lymphoma and 3/7 had transformed follicular lymphoma. These patients included both subsequent responders and non-responders (NR) to a commercial CAR T product (Table S1).

Flow cytometry of thawed CAR T cells showed that HD (*n* = 13) and HD TCR^−^ (*n* = 10) CAR T cells had a higher proportion of naïve and central memory CD8^+^ CAR T cells than lymphoma CAR T cells (*n* = 6) (Fig. 1b), and a higher proportion of CD8^+^CD27^+^PD-1^−^ CAR T cells (HD mean 21.53% vs. lymphoma mean 5.733%, *p* = 0.0464; HD TCR^−^ mean 26.56% vs. lymphoma mean 5.733%, *p* = 0.0103, one-way ANOVA *p* = 0.0182, with Tukey’s multiple comparisons test for paired comparisons) (Fig. 1c). Lymphoma CD8^+^ CAR T cells more frequently co-expressed PD-1 and TIM3, than HD CD8^+^ CAR T cells (Supplementary figure) and had a higher proportion of triple positive CD8^+^CAR T cells expressing PD-1, TIM3 and LAG3 than HD CAR T cells (*p* = 0.039) (Fig. 1e). However, when comparing gene-edited HD TCR^−^ CAR T cells with lymphoma CAR T cells, this difference did not reach statistical significance. Lymphoma CAR T cells had a higher CD4:CD8 ratio than HD TCR^−^ CAR T cells (Supplementary data). In vitro activation assays showed that lymphoma CAR T cells had higher baseline expression of the early activation marker CD69, and less antigen-specific activation upon stimulation with the CD19^+^ NALM-6 cell line (Fig. 1d), which may suggest more differentiated CAR T cells at risk of exhaustion. However, upregulation of CD25, a late activation marker was comparable in all groups (supplementary data). In 24 h in vitro cytotoxicity assays HD, HD TCR^−^ and lymphoma CAR T cells showed similar killing capacity against NALM-6 and Raji CD19^+^ cell lines (Fig. 1f, g). IFN-γ secretion was demonstrated in co-culture of CAR T cells with NALM-6 and was comparable between CAR T groups (Fig. 1h).

**Fig. 2:**
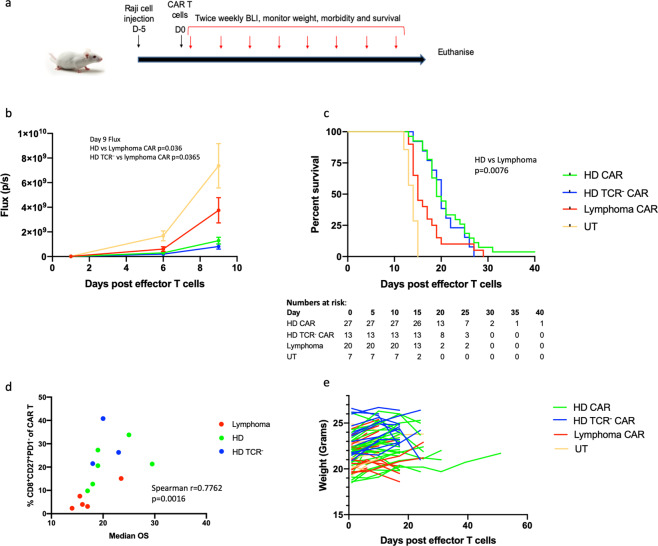
In vivo functional comparison of lymphoma and healthy donor CAR T cells. **a** Schematic diagram of in vivo experiment design with Raji xenograft model. Mice are injected i.v. with 1 × 10^5^ Raji luciferase cells on day −5, on day 0 they are injected i.v. with 5 × 10^5^ CAR^+^ T cells. Tumour growth is monitored twice weekly with BLI following IP injection of luciferin. **b** Raji cell growth is faster in lymphoma CAR T-treated mice compared to HD and HD TCR^−^ CAR T-treated mice. Mean flux and standard error margin is shown for each treatment group with data pooled from two experiments. Day 9 flux HD CAR vs. lymphoma CAR *p* = 0.036, HD TCR^−^ CAR vs. lymphoma CAR *p* = 0.0365, one-way ANOVA *p* ≤ 0.0001 with the Tukey’s multiple comparisons test for paired analysis. **c** Survival curves demonstrate improved survival in HD compared to lymphoma CAR T-treated mice (*p* = 0.0076, log rank test), but comparable survival between HD and HD TCR^−^ CAR T-treated mice. **d** Median overall survival of mice correlated with the proportion of pre-infusion CAR T cells, which were CD8^+^CD27^+^PD-1^−^ (Spearman *r* = 0.7762, *p* = 0.0016) (*n* = 6 HD, *n* = 3 HD TCR^−^ and *n* = 5 lymphoma CAR T products, each tested in 3–5 mice e.g. *n* = 27 HD, *n* = 13 HD TCR^−^ and *n* = 20 lymphoma treated mice). **e** Mice were weighed twice a week during the experiment and results are plotted against time.

An in vivo CAR T-cell ‘stress test’ was performed to see if there were differences in potency between CAR T-cell products not identified by in vitro assays [13]. A subtherapeutic dose of CAR T cells was given to Raji luciferase bearing NSG mice, allowing tumour escape to occur and thereby challenging the CAR T-cell product. NSG mice were injected via the tail vein with 1 × 10^5^ Raji luciferase cells. Five days later, once tumour engraftment was established, mice were injected i.v. with 5 × 10^5^ CAR^+^ T cells from HD (*n* = 6), HD TCR^−^ (*n* = 3) or B-cell lymphoma (*n* = 5) CAR T-cell products. Untransduced T (UT) cells were used as a negative control (Fig. 2a). Cell products from each donor or patient were tested in groups of 3–5 mice (HD *n* = 27, HD TCR^−^ *n* = 13 and lymphoma *n* = 20 mice). Mice were monitored daily for signs of distress by technicians who were not aware of the CAR T group assignment. Tumour growth was assessed twice weekly with bioluminescence imaging, following intraperitoneal luciferin injection. Mice were euthanised when they developed signs of distress persisting for >48 h such as being hunched, or immediately if they developed difficulty moving or breathing.

Flux data demonstrated rapid tumour growth in mice treated with UT cells until day 9 post T-cell injection, after which bioluminescence signal became saturated. Tumour growth was slower in CAR T-treated mice. There was more effective tumour control in HD and HD TCR^−^ CAR T-treated mice than in lymphoma CAR T-treated mice (day 9 flux HD CAR vs. lymphoma CAR *p* = 0.036, HD TCR^−^ CAR vs. lymphoma CAR *p* = 0.0365, one-way ANOVA *p* ≤ 0.0001 with the Tukey’s multiple comparisons test for paired analysis) (Fig. 2b).

UT mice survived a median of 14 days (range 12–15 days) post T-cell injection. Survival was increased in all CAR T-cell groups. However, survival was longer in HD (median 19 days, range 13–72 days) and HD TCR^−^ (median 20 days, range 14–27) CAR T-treated mice compared to the lymphoma CAR T group (median 15 days, range 13–29) (log rank test, HD vs. lymphoma CAR T *p* = 0.0076, HD TCR^−^ vs. lymphoma *p* = 0.0859) (Fig. 2c). In paired analysis of HD and HD TCR^−^ CAR T cells from the same HDs (*n* = 3), each tested in 3–5 mice, no difference was seen in survival (HD median survival 20 days, range 13–27, HD TCR^−^ median survival 20 days, range 14–27 days), suggesting that gene-editing to remove TCR expression did not impair T-cell function.

HD CAR T-cell products could potentially allow the selection of ‘good performers’, thus increasing the functional advantage seen with HD CAR T cells. Differences were seen in performance between individual lymphoma patient and HD-derived CAR T-cell products. In order to identify the characteristics of a superior CAR T-cell product, we correlated median survival with CAR T-cell phenotypes (Spearman’s correlation). There was no correlation with the proportions of CD45RO^−^CCR7^+^ naïve and stem cell memory, CD45RO^+^CCR7^+^ central memory, CD45RO^+^CCR7^−^ effector memory or CD45RO^−^CCR7^−^ terminal effector CAR T cells, on either CD4 or CD8 CAR T cells. Similarly, there was no correlation with percentage of CD8 or CD4 CAR^+^ T cells expressing PD-1, LAG3 or TIM3. However, the percentage of CAR T cells, which were CD8^+^CD27^+^PD-1^−^, thought to represent naïve, stem cell memory and central memory populations [5], significantly correlated with median survival (Spearman *r* = 0.7762, *p* = 0.0016) (Fig. 2d).

It has been shown that product-related T-cell characteristics influence clinical outcome, for example in CLL the proportion of a specific memory population (CD8^+^CD27^+^PD-1^−^) correlated with remission status [5] and in B-cell lymphoma CD8^+^CCR7^+^CD27^+^ cells were three times higher in the infusion product from patients who achieved a CR compared to those who did not [14]. We found a higher proportion of this CD8^+^CD27^+^PD-1^−^ population in HD and HD TCR^−^ CAR T cells compared with lymphoma CAR T cells. Although, the functionality of this population has only been reported in CLL, a recent paper, which examined single-cell transcriptomics and clonal evolution of CAR T cells found a preferential expansion of CAR^+^CD8^+^CD27^+^ clones infused into lymphoma patients, using a different CAR construct and manufacturing process [15]. Furthermore, Fraietta et al. demonstrated that removal of CAR^+^ CD8^+^CD27^+^PD-1^−^ T cells resulted in loss of tumour control in the NALM-6 NSG xenograft model [5]. Using a different tumour model, CAR construct and T-cell donor source, we have shown that the proportion of CAR^+^CD8^+^CD27^+^PD-1^−^ cells from HDs and lymphoma patients correlated with median survival of mice in a Raji NSG xenograft model, thus underlining the significance of this population. 4/6 lymphoma patients, tested in our model, achieved a CR at 1 month post infusion, and in this group, there was a trend towards a higher proportion of CAR^+^CD8^+^CD27^+^PD-1^−^ T cells (mean 7.435% vs. 2.735% in NR patients), but the numbers were too small to draw conclusions, furthermore at 3 months only one patient remained in CR.

We did not specifically select HDs with a high percentage of CAR^+^CD8^+^CD27^+^PD-1^−^ T cells. However, an ‘off-the-shelf’ HD TCR^−^ CAR T-cell product would enable screening for products with a high proportion of this population. The HDs in this study were all young adults. It is not known whether the inferior performance of autologous CAR T cells derived from lymphoma patients was the result of T-cell senescence, prior therapies or tumour-induced dysfunction. It is therefore not possible to determine from our data whether younger lymphoma patients and those who have received fewer lines of therapy would also have an inferior CAR T product compared to HD CAR T cells. The use of age-matched controls and untreated lymphoma patients would provide data unaffected by cell senescence or treatment-induced dysfunction, but our comparison is clinically relevant and representative of lymphoma patients currently accessing commercial CAR T-cell products.

Other groups have shown superior function of HD CAR T cells compared to patient-derived CAR T cells in vivo [5, 6], but these HD CAR T products were not suitable for treating patients due to the risk of GvHD. This study has shown for the first time in a pre-clinical model the superior functionality of a HD TCR^−^ CAR T-cell product compared to lymphoma patient-derived CAR T cells. Further work is needed to identify the optimal donor for an ‘off-the-shelf’ allogeneic product. In order to maximise the therapeutic benefit of HD TCR^−^ CAR T cells, host rejection of non-HLA-matched CAR T cells would need to be safely overcome.

## Supplementary information

Supplementary Table

Supplementary Figure
