# Supplementary figures and images for: Gene-edited healthy donor CAR T cells show superior anti-tumour activity compared to CAR T cells derived from patients with lymphoma in an in vivo model of high-grade lymphoma
Source: Leukemia. 2021 Jun 18;35(12):3581–4. doi: 10.1038/s41375-021-01324-z (PMC8632681; doi:10.1038/s41375-021-01324-z)

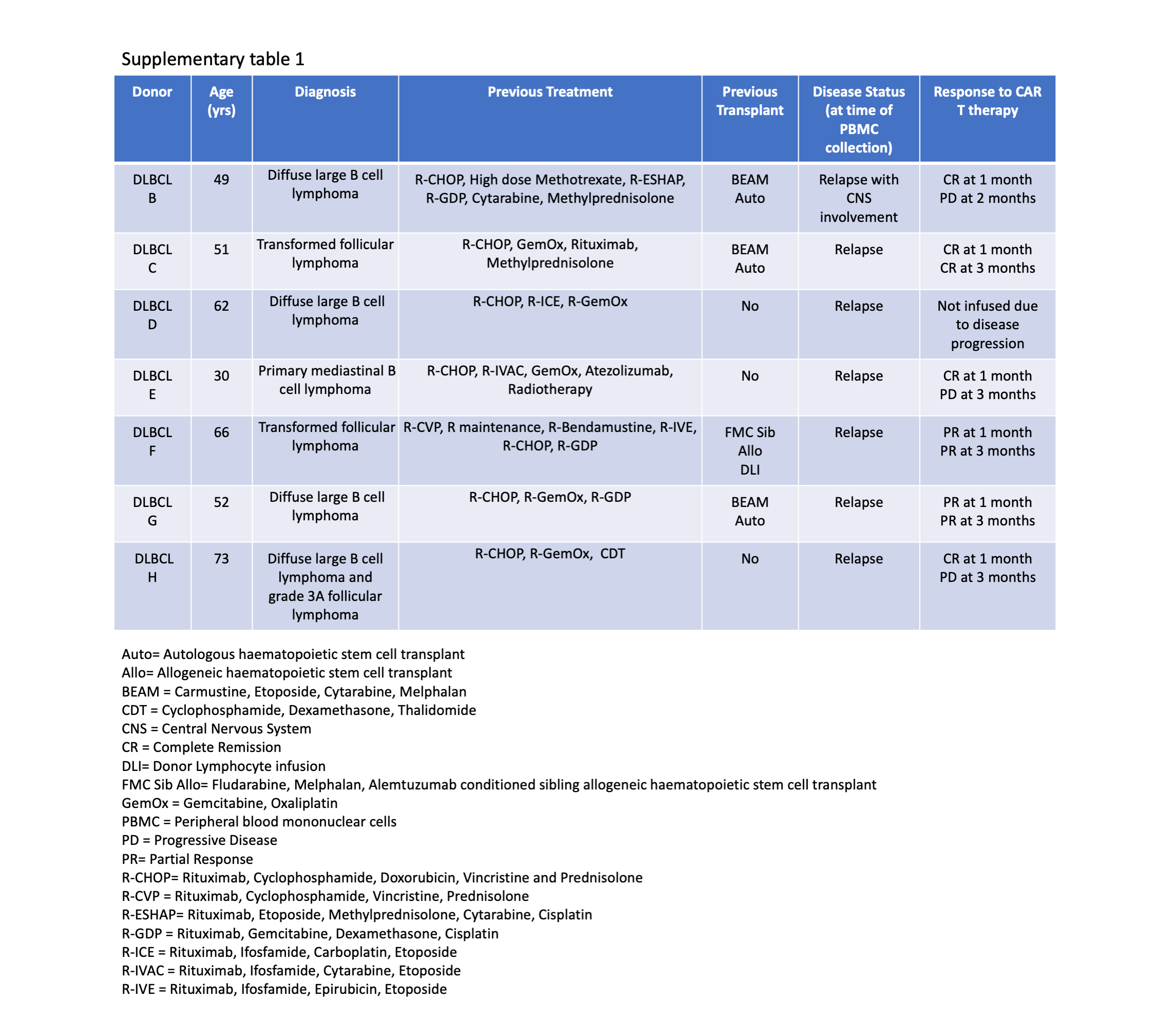

Supplement: Supplementary file 1 — Supplementary Table [file 41375_2021_1324_MOESM1_ESM.tif]

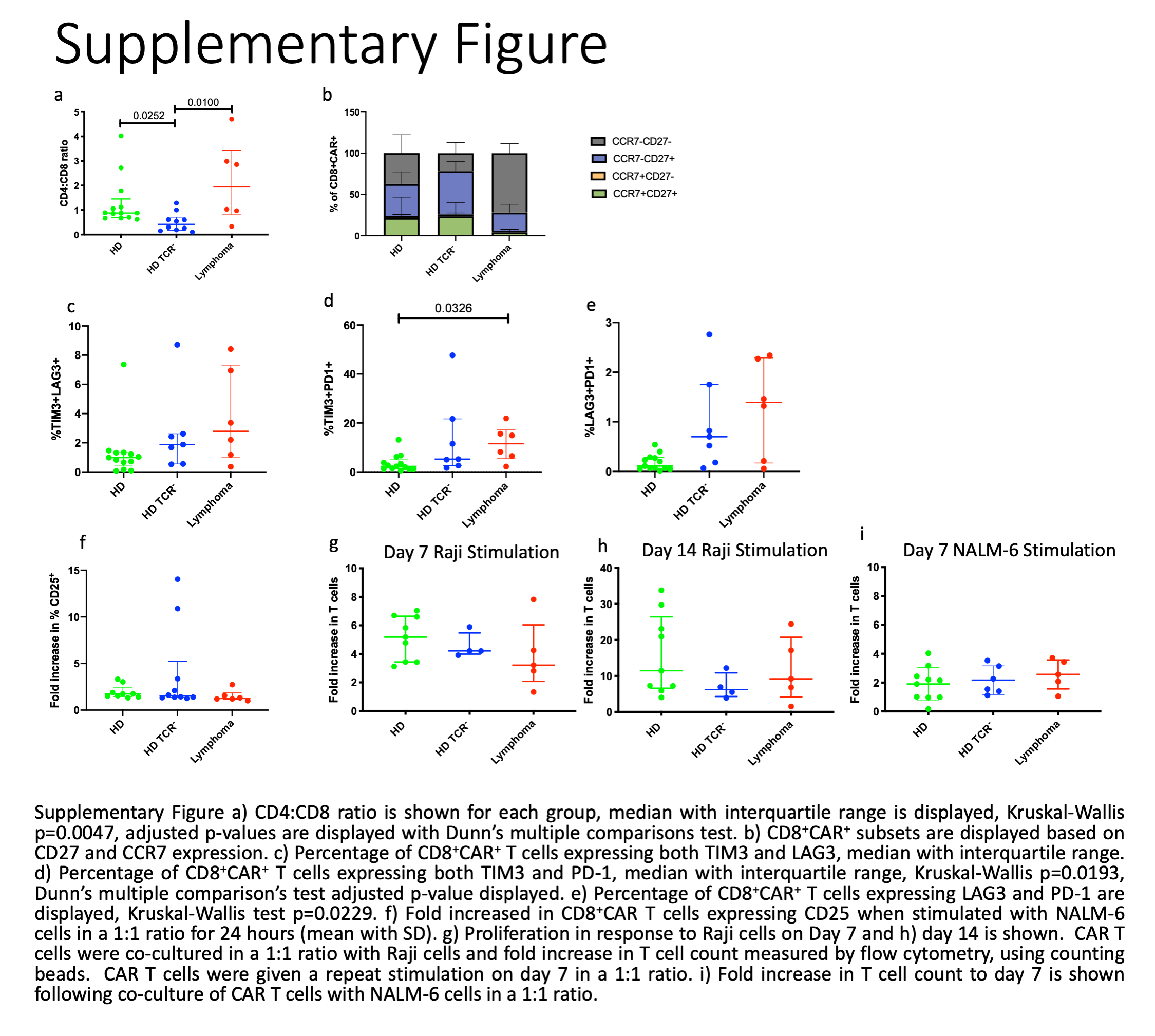

Supplement: Supplementary file 2 — Supplementary Figure [file 41375_2021_1324_MOESM2_ESM.tif]
